# Spectrophotometric determination of phenol impurity in phenoxyethanol and phenol index of drinking water and municipal wastewater effluent after salting-out assisted liquid phase microextraction (SA-LPME)

**DOI:** 10.1016/j.heliyon.2024.e27143

**Published:** 2024-02-27

**Authors:** Farideh Roustaei, Majid Baghdadi, Azam Marjani, Mohammad Alimoradi

**Affiliations:** a Department of Chemistry, Faculty of Sciences, Islamic Azad University, Arak Branch, Arak, Iran; b Department of Environmental Engineering, Graduate Faculty of Environment, University of Tehran, P.O. Box: 1417853111, Tehran, Iran

**Keywords:** Phenol, Phenol index: 2-phenoxyethanol, Salting-out assisted liquid phase microextraction

## Abstract

In this study, a novel and convenient analytical method based on salting-out-assisted liquid phase microextraction (SA-LPME) has been developed. A spectrophotometric technique was employed to quantify the concentration of phenol in drinking water and treated wastewater, as well as the phenol impurity in 2-phenoxyethanol (PE). To accomplish this, a solution containing dissolved PE was supplemented with 4-aminoantipyrine (4-AAP) and hexacyanoferrate. Subsequently, NaCl was added to induce the formation of a two-phase system, consisting of fine droplets of PE as an extractant phase in the aqueous phase. The resulting red derivative was then extracted into the extractant phase and separated through centrifugation. Finally, the absorbance of the extracted derivative was measured at 520 nm. The Response Surface Methodology (RSM) based on the Box-Behnken Design (BBD) was employed to optimize the influential factors, namely 4-Aminoantipyrine (4-AAP), buffer (pH = 10), hexacyanoferrate, and NaCl. By utilizing the optimal conditions (buffer: 50 μL, 4-AAP (1% w/v): 80 μL, hexacyanoferrate (10% w/v): 65 μL, and NaCl: 0.7 g per 10 mL of the sample), the limit of detection was determined to be 0.7 ng mL^−1^ and 0.22 μg g^−1^ for water and PE samples, respectively. The relative standard deviation (RSD) and correlation of determination (r^2^) obtained fell within the range of 2.4–6.8% and 0.9983–0.9994, respectively. Moreover, an enrichment factor of 65 was achieved for a sample volume of 10 mL. The phenol concentration in two PE samples (PE-1, PE-2), provided by a pharmaceutical company (Pars Sadra Fanavar, Iran), were determined to be 0.83 ± 0.05 μg g^−1^ and 2.70 ± 0.14 μg g^−1^, respectively. Additionally, the phenol index in drinking water and treated municipal wastewater was found to be 3.60 ± 1.06 ng mL^−1^ and 4.60 ± 1.17 ng mL^−1^, respectively. These mentioned samples were spiked in order to evaluate the potential influence of the matrix. The relative recoveries from PE-1, PE-2 samples, drinking water, and treated municipal wastewater samples were measured as 104.5%, 97.5%, 101.6%, and 107.8%, respectively, indicating no matrix effect.

## 1 Introduction

Phenolic compounds are considered hazardous contaminants that are released into the environment as a byproduct of various human activities, such as the production of plastics, pharmaceuticals, insecticides, textiles, paper, and dyes [1]. These compounds possess harmful effects on both living organisms and the environment, even when present in low concentrations. Consequently, the European Community (EC) has classified them as priority compounds [2,3]. The Phenol Index (PhI) is a measure of the overall content of phenolic compounds in water, thereby providing an indication of the extent of phenolic contamination [4]. In order to regulate the presence of phenolic compounds in drinking water, the EC has implemented legal thresholds of 0.5 μg L^−1^ for the total quantity of phenolic compounds and 0.1 μg L^−1^ for each individual phenolic compound [5,6].

With the growing usage of chemicals in society, the quality of water sourses is increasingly threathed by a rising prevalence of aquatic micropollutants. This emergence of micropollutants in surface water resources has been primarily attributed to the discharge of effluent from wastewater treatment plants. Although these plants partially eliminate micropollutants, their discharge into surface water remains a significant contributing factor to the occurrence of micropollutants [7,8]. Additionally, conventional water treatment processes, originally designed for pathogen and nutrient removal, have exhibited limited efficacy in removing micropollutants. Consequently, the insufficient removal of micropollutants in conventional drinking water treatment facilities may result in human exposure [9].

The presence of process-related impurities in pharmaceutical drug substances refers to the existence of unwanted chemicals in active pharmaceutical compounds. These impurities can be generated by various factors, such as the manufacturing process itself and unsuitable storage conditions [10]. Raw materials, intermediates, reagents, and residual components that arise during the production of active pharmaceutical ingredients (API) may remain as impurities in the final product. This occurrence is inevitable, as no chemical reaction can be completed [11]. Even trace quantities of these undesirable chemical constituents can have an impact on the safety and efficacy of a drug. As a result, regulatory authorities have recently placed significant emphasis on impurity profiles in both manufactured medicinal products and pharmaceutical active ingredients [2].

2-Phenoxyethanol (PE) is an aromatic ether compound extensively employed as a preservative within the cosmetics industry [12]. The production process involves the reaction of phenol with ethylene oxide, facilitated by a basic catalyst, under conditions of elevated temperature and pressure [13]. However, the presence of phenol impurity in PE has always been a problem due to the adverse effects of phenolic compounds on human health. Consequently, the accurate detection of phenol in PE is of utmost importance to mitigate any potential impacts on consumers.

The spectrophotometric determination of phenol was revolutionized by Emerson in 1943 through the development of the 4-aminoantipyrine (4-AAP) derivatization method for phenolic compounds. This approach is widely utilized for assessing the Phenol Index due to its notable advantages such as high efficiency, cost-effectiveness, and rapid reaction kinetics [8].

The methodology is based on the oxidative coupling of phenolic compounds with 4-AAP in the presence of an oxidant, leading to the formation of antipyrine dyes. In this technique, 4-AAP reacts with unsubstituted, meta-substituted, or ortho-substituted phenolic compounds containing various functional groups such as halogen or sulfonate, methoxy, or carboxy groups. The reaction occurs at a pH of 10.0 and yields persistent red-colored antipyrine dyes [14]. These antipyrine dyes enable the subsequent spectrophotometric determination of phenolic compounds [15].

Despite the significant advancements in analytical methods in recent decades, sample preparation is still required before conducting chemical analysis. Its primary objective is to concentrate the analyte, clean up the extract, and enhance the signal. Among various techniques, liquid-phase microextraction (LPME) technologies with diverse geometries are considered promising methods for sample preparation [16].

Dispersive liquid-phase microextraction (DLPME) has garnered significant attention among microextraction techniques. This method involves the dispersion of fine droplets of extraction solvent within an aqueous sample. Moslemzadeh et al. reported a procedure based on the reaction of phenol with 4-aminoantipyrine (4-AAP) in the presence of an oxidizing agent, resulting in the generation of an extractable compound through ultrasound-assisted dispersive liquid–liquid microextraction (UA-DLLME). Subsequently, the determination of phenol was carried out utilizing a smartphone-based colorimetric system [5]. Zahedi et al. detailed the utilization of dispersive liquid–liquid microextraction for the preconcentration of trace phenolic compounds in artificial seawater subsequent to derivatization with 4-aminoantipyrine. The volume ratio of 11:1 was achieved for the dispersion to the organic phase [17]. One notable drawback of DLLME is the usage of a substantial quantity of hazardous dispersing organic solvents, known to have an adverse impact on extraction recovery [18]. In a separate study, Hu et al. presented dispersive liquid-liquid microextraction using a novel hydrophobic deep eutectic solvent for the determination of phenolic compounds. This investigation involved the synthesis of a new terpineol-based hydrophobic deep eutectic solvent by combining 1-octanoic acid with α-terpineol [19]. Despite the relatively low toxicity of deep eutectic solvents, their limited commercial availability restricts their routine utilization for analyses [20].

One of the most prevalently recognized homogeneous extraction methods is salting-out-assisted liquid phase microextraction (SA-LPME). This process involves the addition of a significant quantity of salt to an aqueous solution containing an organic solvent. Consequently, the solubility of the organic phase diminishes, resulting in the generation of small droplets of the extractant phase within the solution. Notably, this technique does not necessitate the use of a dispersing solvent. Owing to its compatibility with a diverse array of analytical techniques, such as gas chromatography [21], UV–vis spectrophotometry [22], high-performance liquid chromatography, and numerous other methods, it has gained wide applicability. This method is particularly advantageous as it makes use of water-soluble organic solvents. Its successful application spans across a variety of matrices, including water [23], milk [24], fruit juice [25], urine [26], serum [27], and plasma [28].

Due to the potential toxicity associated with phenolic compounds, it is essential to develop innovative, rapid, and reliable quantitative methodologies for the determination of trace amounts of these compounds. The primary objective of this study is to develop a fast, user-friendly, and cost-effective salting-out assisted liquid phase microextraction (SA-LPME) approach, coupled with spectrophotometric detection, aimed at identifying the presence of the phenol impurity in 2-phenoxyethanol, as well as the phenol index in samples of drinking water and treated municipal wastewater. The impact of buffer, potassium hexacyanoferrate, 4-AAP, and NaCl was meticulously examined to optimize the extraction efficiency of the SA-LPME method. After the derivatization of phenol with 4-AAP, the resultant antipyrine dye facilitated the spectrophotometric determination of phenol. At present, to the best of our knowledge, no studies have been reported on the application of the SA-LPME technique for detecting phenol in 2-phenoxyethanol employing the aminoantypirine method. Noteworthily, this technique capitalizes on the use of 2-phenoxyethanol as the extraction solvent, thereby streamlining the process and rendering it cost-effective.

## 2 Materials and methods

### 2.1 Materials and instruments

All chemicals employed in this investigation met the standards of analytical reagent grade. The 2-Phenoxyethanol samples were obtained from the pharmaceutical company Pars Sadra Fanavar in Iran. Moreover, 2-phenoxyethanol (C_6_H_5_OCH_2_CH_2_OH, CAS: 122-99-6, purity: 99%, synthesis grade) served as the extraction phase for the determination of the phenol index, was acquired from Merck KGaA, Germany. Moreover, 4-aminoantipyrine (C_11_H_13_N_3_O, CAS: 83-07-8, purity: 99%, synthesis grade), methanol (CH₃OH, CAS: 67-56-1, purity: 99.9%, analysis grade), potassium hexacyanoferrate (K3Fe(CN)6, CAS: 13,746-66-2, purity: 99%, analysis grade), sodium sulfate (Na_2_SO_4_, CAS: 7757-82-6, purity: 99%, analysis grade), and sodium chloride (NaCl, CAS: 7647-14-5, purity: 99.5%, analysis grade), all obtained from Merck KGaA, Germany. Additionally, a buffer solution (pH = 10, Product code: 42,449) was purchased from Hach Company, Germany [29].

A pH meter (AD1000, Adwa, Hungary) was employed for measuring and adjusting the pH of the solutions. The preparation of stock solutions of 4-AAP (1% w/v) and potassium hexacyanoferrate (10% w/v) was conducted by the dissolution of the appropriate quantities of the respective chemicals in methanol and water, respectively. Prior to conducting the salting-out assisted liquid phase microextraction procedure on samples of drinking water and treated wastewater, the purification of 2-phenoxyethanol was carried out by washing with a sodium hydroxide solution (1 mol/L) three times, followed by rinsing with deionized water. Subsequently, it was dried using anhydrous sodium sulfate and later stored at 4 °C. The absorbance of the resultant antipyrine dyes was measured using a UV/Vis spectrophotometer (HACH-DR5000, Germany) at a wavelength of 520 nm. To expedite the separation process, a centrifuge (Hitachi, Japan) was utilized.

### 2.2 Phenoxyethanol and drinking water samples

The 2-phenoxyethanol samples were stored under refrigeration conditions. Drinking water samples were obtained from the tap water source at the University of Tehran, Iran. Concurrently, treated municipal wastewater samples were provided from the effluent of the final clarifier unit, before the chlorination unit, within the Ekbatan wastewater treatment plant. Ten individual samples (1000 mL) were collected per hour. After mixing the collected samples, 500 mL of the resultant homogeneous sample was filtered through a 0.45 μm filter. All water and treated municipal wastewater samples were collected in glass containers, with their respective pH levels being adjusted to 4 or lower through the application of a 10% phosphoric acid solution. The preserved samples were stored at, or below, 6 °C for 24 h.

### 2.3 Salting-out assisted liquid phase microextraction procedure

A conical bottom tube was utilized for the sequential addition of 10 mL of ultrapure water, followed by the dissolution of 250 μL of the 2-phenoxyethanol sample. Subsequently, 50 μL of buffer (pH = 10) and 80 μL of the 4-AAP solution were introduced to the prepared sample and shaken. Following this, 65 μL of potassium hexacyanoferrate solution was added and agitated. Thereafter, 0.7 g of NaCl was introduced, followed by vigorous agitation for a period of 30 s, thereby resulting in a cloudy solution containing fine droplets of 2-phenoxyethanol. Ultimately, the cloudy solution was centrifuged to sediment the extractant phase containing the produced antipyrine dyes. The supernatant solution was subsequently decanted, and 70 μL of methanol was introduced to the sedimented phase to mitigate its viscosity. The diluted phase was then transferred to a microliter cell, and its absorbance was measured at 520 nm.

In the execution of the salting-out assisted liquid phase microextraction procedure for the assessment of the phenol index in drinking water and treated municipal wastewater samples, 250 μL of purified 2-phenoxyethanol, serving as the extraction phase, was dissolved in the samples. The other steps of the process were conducted similarly to the procedure described above in this section.

### 2.4 Experimental design

The aim of the experimental design is to determine the optimal values for the variables by analyzing the effect of factors on the response and assessing parameter interactions [30]. Within this study, the Box-Behnken Design (BBD) was employed, operated by the Design Expert Software Version 12.0.3.0, to design experiments and evaluate the influence of factors affecting the response. These factors include buffer (A), 4-AAP (B), potassium hexacyanoferrate (C), and NaCl (D). The following equation was used within the BBD for determining the number of required experiments (N) [31].

(1) $$N=2k\left(k-1\right)+C_{0}$$

Where the number of central points is *C*_*0*_, and the variable number is *k*.

The relationship between the studied variables and the response is demonstrated using the second-order polynomial equation (Eq. 2), which is derived from an incomplete factorial design.

(2) $$Y=\sum \beta_{i}X_{i}+\sum \beta_{ii}{X_{i}}^{2}+\sum \beta_{ij}X_{i}X_{j}+\beta_{0}+\varepsilon$$

In this equation, X_i_ denotes the influence of the variables, while Y represents the response. X_i_ X_j_ signifies the interaction term, and X_i_^2^ denotes the quadratic terms. Furthermore, the terms βi, βii, and βji (j≠i) correspond to the coefficients of linear, quadratic, and interaction, respectively. *ε* represents the constant error, and β0 signifies the random error [32].

The software design proposed a matrix design comprising 29 experiments. High and low levels for each parameter were determined based on preliminary experiments (Table 1). The model was evaluated using Analysis of Variance (ANOVA), and response surfaces were generated for optimization purposes. The significance of the model was assessed by considering the p-value obtained from the ANOVA analysis at a 95% confidence level.

**Table 1:** The coded and real levels of the input parameters.

Variables	Unit/10 mL	Levels		
		Low	Central	High
		−1	0	1
A: Buffer	μL	5	77.5	150
B: 4-AAP	μL	5	52.5	100
C: Hexacyanoferrate	μL	5	52.5	100
D: NaCl salt	g	0.2	0.5	0.8

## 3 Result and discussion

### 3.1 Statistical analysis

The experimental conditions outlined by the Box-Behnken Design (BBD) and the resultant experimental findings are presented in Table 2. The following quadratic equation, formulated through the BBD, provides the correlation between the influential parameters and the absorbance:

Y = 0.007972 A + 0.001376 B + 0.008655 C + 2.79283 D - 0.000021 A × B - 0.000059 A × C - 0.003494 A × D + 0.000020 B × C + 0.010298 B × D - 0.000018 A^2^ - 0.000021 B^2^ - 0.000041C^2^ - 2.53991 D^2^

Where A, B, C, and D are the amounts of buffer, 4-AAP, the hexacyanoferrate, and NaCl, respectively, and Y is absorbance.

**Table 2:** The proposed experiments according to the Box-Behnken Design and the resulting absorbance for each experiment.

Run	A: Buffer	B: Aminoantipyrine	C: Hexacyanoferrate	D: Salt	Absorbance
	(μL)	(μL)	(μL)	(%)	
1	5.0	52.5	100.0	0.5	0.597
2	77.5	5.0	100.0	0.5	0.187
3	5.0	52.5	5.0	0.5	0.100
4	77.5	100.0	52.5	0.2	0.083
5	77.5	100.0	5.0	0.5	0.449
6	77.5	100.0	100.0	0.5	0.626
7	77.5	5.0	5.0	0.5	0.195
8	5.0	5.0	52.5	0.5	0.156
9	77.5	52.5	100	0.2	0.079
10	77.5	100.0	52.5	0.8	0.700
11	77.5	5.0	52.5	0.2	0.072
12	150	100.0	52.5	0.5	0.480
13	77.5	52.5	5.0	0.2	0.042
14	150.0	5.0	52.5	0.5	0.180
15	77.5	52.5	52.5	0.5	0.556
16	150.0	52.5	52.5	0.2	0.020
17	5.0	52.5	52.5	0.2	0.020
18	77.5	52.5	52.5	0.5	0.541
19	150	52.5	52.5	0.8	0.191
20	77.5	52.5	52.5	0.5	0.480
21	5.0	100.0	52.5	0.5	0.740
22	150	52.5	100.0	0.5	0.150
23	77.5	52.5	100.0	0.8	0.402
24	77.5	5.0	52.5	0.8	0.102
25	5.0	52.5	52.5	0.8	0.495
26	77.5	52.5	52.5	0.5	0.431
27	150	52.5	5.0	0.5	0.467
28	77.5	52.5	5.0	0.8	0.308
29	77.5	52.5	52.5	0.5	0.570

The Analysis of Variance (ANOVA) was utilized to evaluate the significance of the model and the associated terms. The results of the ANOVA can be found in Table 3. An F-value of 47.38 and a p-value of less than 0.0001 for the model signify its significance, thus indicating that the equation aptly represents the relationship between the response and the parameters. Furthermore, the obtained p-value for the lack of fit (LOF) was recorded as 0.8015, suggesting that other factors within the experiments only induced a minimal level of interference, underscoring the precise alignment of the model with the data in this study [33].

**Table 3:** The Analysis of variance (ANOVA) for the quadratic model.

Source	Sum of Squares	df	Mean Square	F-value	p-value	
**Model**	1.42	13	0.1090	47.38	<0.0001	significant
A-Buffer	0.0320	1	0.0320	13.93	0.0020	
B-4-AAP	0.3982	1	0.3982	173.15	<0.0001	
C-Hexacyanoferrate	0.0192	1	0.0192	8.35	0.0112	
D-Salt	0.2952	1	0.2952	128.34	<0.0001	
AB	0.0202	1	0.0202	8.77	0.0097	
AC	0.1656	1	0.1656	72.03	<0.0001	
AD	0.0231	1	0.0231	10.05	0.0063	
BC	0.0086	1	0.0086	3.72	0.0729	
BD	0.0861	1	0.0861	37.46	<0.0001	
A^2^	0.0565	1	0.0565	24.57	0.0002	
B^2^	0.0141	1	0.0141	6.12	0.0258	
C^2^	0.0556	1	0.0556	24.18	0.0002	
D^2^	0.3389	1	0.3389	147.38	<0.0001	
**Residual**	0.0345	15	0.0023			
Lack of Fit	0.0208	11	0.0019	0.5546	0.8015	not significant
Pure Error	0.0137	4	0.0034			
**Cor Total**	1.45	28				

R^2^ = 0.9762, adjusted R^2^ = 0.9556, predicted R^2^ = 0.907.

All of the examined variables listed in Table 3 exhibited a p-value below 0.05, validating their influence on the response. Notably, 4-AAP demonstrated a substantial impact on the microextraction process, evidenced by an F-value of 173.15 and a p-value below 0.0001. Similarly, the salt concentration, with a p-value of less than 0.001, exhibited a significant effect on the microextraction process.

The regression equation derived from the ANOVA analysis yielded a coefficient of determination (R^2^) value of 0.9556, indicating the proportion of variation accounted for by the model. Consequently, the model can explain 95.56% of the total variance in the response.

Furthermore, the calculated value for the adjusted R^2^ was 0.9665, signifying a strong relationship between the model and the experimental data. Additionally, the predicted R^2^ was determined to be 0.9077, highlighting the model's high potential in predicting the response. The high values of R^2^ affirm the model's applicability in analyzing and optimizing the influences of the studied parameters on the absorbance in the microextraction procedure [34,35].

**Fig. 1:**
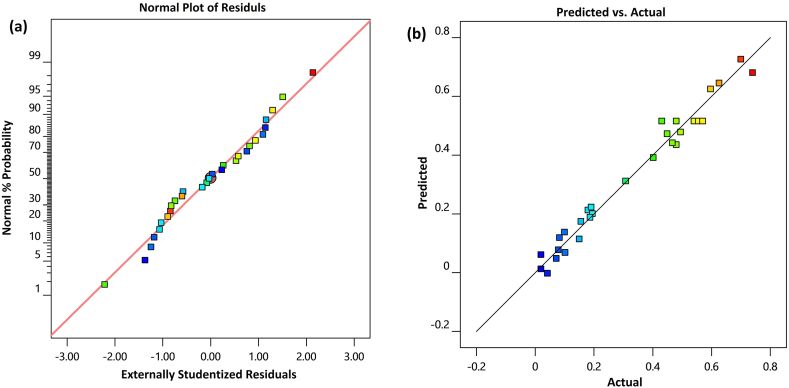
(a) Normal probability plot of standardized residuals, (b) The predicted responses versus the observed responses obtained by the BBD.

Fig. 1(a) depicts the normal residual plot, confirming the model's adequacy. Furthermore, Fig. 1(b) presents the actual values versus predicted responses, illustrating that most of the points are dispersed along the sloping line. This correlation between actual and predicted responses indicates the excellent fit of the quadratic model to the experimental data.

### 3.2 Impact of independent variables on the absorbance

Phenolic compounds undergo a reaction with 4-aminoantipyrine in the presence of an oxidant agent, such as hexacyanoferrate, in an alkaline medium, resulting in the formation of a colored product. Consequently, any changes in the concentrations of 4-AAP and hexacyanoferrate directly impact the intensity of the color produced. Moreover, the absorbance is directly linked to the extraction recovery, which is influenced by the volume of the extractant phase. Additionally, the volume of the extractant phase is significantly influenced by the quantity of water-miscible organic phase and the concentration of salt. Increasing the salt concentration reduces the solubility of the solvent, thereby leading to an increase in the volume of the extractant phase [5,36].

**Fig. 2:**
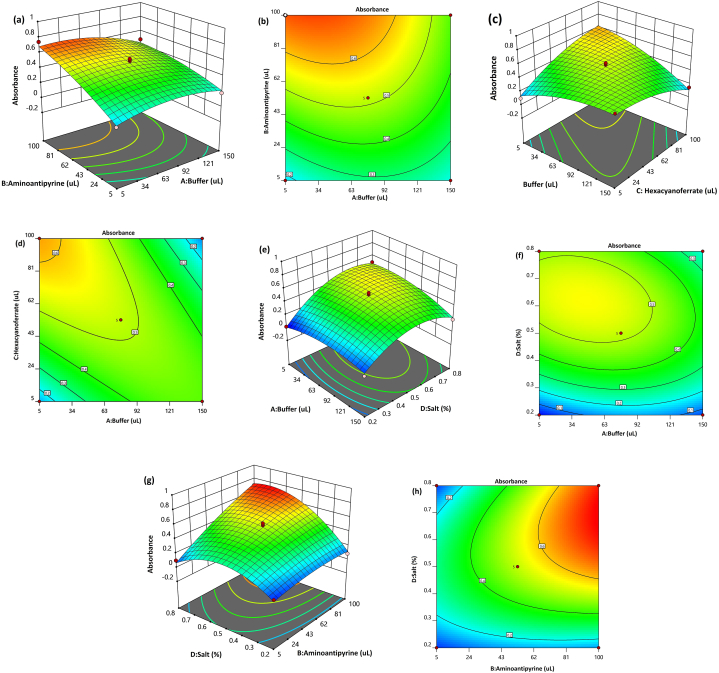
(a) 3D response surface and (b) contour plot for buffer and 4-AAP, (c) 3D response surface and (d) contour plot for buffer and hexacyanoferrate, (e) 3D response surface and (f) contour plot for buffer and salt, (g) 3D response surface and (h) contour plot for 4-AAP and salt.

The software-generated 3D response surfaces and contour plots provide valuable insights into the influence of independent variables (buffer, 4-AAP, hexacyanoferrate, and NaCl) on absorbance. These graphical representations illustrate the impact of two variables on the response while keeping the other factors at their central values. They effectively demonstrate the relationship between the response and the levels of these two parameters. Specifically, Fig. 2(a) and Fig. 2(b) depict the 3D surface response and contour plot, respectively, showing the influence of 4-AAP and buffer on absorbance. The absorbance exhibited an upward trend as the amount of 4-AAP increased from 5 to 100 μL, attributed to the formation of a greater quantity of antipyrine dye. This behavior aligns with findings from other studies in the literature [17] regarding the impact of 4-AAP on phenol extraction.

It has been reported that the pH range from 9.8 to 10.2 is suitable for the color development reaction [37]. To achieve the desired pH, a buffer solution with a pH of 10 was employed. It is evident that when the buffer volume was below 90 μL, a higher absorbance was observed. Conversely, an increase in buffer volume led to a decrease in absorbance, likely due to a deviation from the pH of 10.0 caused by a higher concentration of salt, which significantly affected the ionic strength.

The influence of the buffer and hexacyanoferrate can be observed in Fig. 2(c) and (d), demonstrating that an increase in hexacyanoferrate resulted in an elevated absorbance due to its crucial role in color development. As depicted in Fig. 2(e) and (f), the absorbance increased up to a certain point with the addition of NaCl. Logically, adding NaCl during the microextraction procedure can enhance the extraction yield by increasing the volume of the extractant phase through the salting-out effect [17].

The introduction of NaCl induces a reduction in the solubility of 2-phenoxyethanol, leading to the formation of the extraction phase initially. This process contributes to an increase in absorbance until the point of complete extraction. However, beyond this stage, the addition of NaCl merely enlarges the volume of the extraction phase, causing a dilution of the extractant phase and subsequently decreasing the absorbance. The 3D surface and contour plot of the salt and 4-AAP concentration, as depicted in Fig. 2(g) and (h), respectively, illustrate the effects of these variables, which have been discussed earlier.

By examining the interaction of the factors presented in Fig. 2, the optimal values for the studied parameters were determined to be 50 μL of buffer, 80 μL of 4-AAP, 65 μL of hexacyanoferrate, and 0.70 g of NaCl per 10 mL of sample. These settings resulted in an absorbance of 0.78.

### 3.3. figures of merit

3.3

The analytical characteristics of the SA-LPME technique were evaluated under the optimized conditions. Table 4 presents the obtained figures of merit, including the enrichment factor (EF), relative standard deviation (RSD), correlation of determination (r^2^), limit of detection (LOD), and other relevant findings. For drinking water samples, the dynamic range (DR) of the calibration curve spanned from 0.2 to 200 ng mL^−1^, while for PE samples, it ranged from 0.7 to 8.0 μg g^−1^. The limit of detection was determined to be 0.7 ng mL^−1^ and 0.22 μg g^−1^ for drinking water and PE samples, respectively, based on the standard deviation of the blank signals and the slope of the calibration curve after preconcentration (3Sb/m). The relative standard deviation (RSD) ranged from 2.4% to 6.8%, and the resulting correlation of determination (r2) ranged from 0.9983 to 0.9994. The enrichment factor was calculated by dividing the slope of the calibration curve after preconcentration by the slope of the calibration curve without preconcentration, yielding a value of 65.

**Table 4:** Analytical performance of the SA-LPME technique.

Sample	Slope	Intercept	r^2^	SD	RSD^1^ (%) (n = 7)	LOD^2^ (μg g^−1^)	DR	
PE-1^4^	0.1689	0.1391	0.9991	0.06	6.8	0.22	0.7–8.0	
PE-2^4^	0.1891	0.5115	0.9983	0.14	5.1	0.24	0.7–8.0	
Sample	Slope	Intercept	r^2^	SD	RSD^a^ (%) (n = 7)	LOD^b^ (ng mL^−1^)	DR	EF^3^
WS with SA-LPME	0.0068	0.1385	0.9994	0.07	2.4 (50)	0.7	2–200	65
WS without SA-LPME	1.046 × 10^−4^	0.0133	0.9995	0.05	2.1 (4000)	112	350–15000	–

1 The phenol concentrations used for the RSD determination are shown in parentheses.

2 Calculated as 3 S_b_/m (*m* and *S*_*b*_ are the slope of the calibration plot and the standard deviation of the blank signal).

3 Calculated as the ratio of the slopes of the calibration graphs produced with and without preconcentration.

4 Two ^4^PE samples (PE-1, PE-2) were provided by a pharmaceutical company (Pars Sadra Fanavar) in Iran.

### 3.4 Analysis of drinking water, treated municipal wastewater, and PE samples

The SA-LPME technique developed in this study was employed to extract and quantify phenol in 2-phenoxyethanol, as well as determine the phenol index in drinking water and treated municipal wastewater samples, in order to validate the proposed method. The results of the analysis of real samples, along with the relative recoveries of spiked samples, are presented in Table 5. For the extraction procedure, a sample volume of 10 mL was used for drinking water samples, while 250 μL of PE dissolved in deionized water was subjected to the extraction process. The tests were carried out under optimal conditions using the method described. The PE samples were analyzed using the standard addition method, showing phenol concentrations of 0.83 ± 0.05 μg g^−1^ and 2.70 ± 0.14 μg g^−1^. Additionally, the phenol index in drinking water and treated municipal wastewater was found to be 3.60 ± 1.06 ng mL^−1^ and 4.60 ± 1.17 ng mL^−1^, respectively. These mentioned samples were spiked to evaluate the matrix effect, and the relative recoveries from the PE-1, PE-2, drinking water, and treated wastewater samples were determined to be 104.5%, 97.5%, 101.6%, and 107.8%, respectively, indicating the absence of a matrix effect.

**Table 5:** Analysis of real samples and the relative recoveries of spiked samples.

Sample	Concentration (mean ± SD^a^)	Added	Founded (mean ± S.D^a^ (	Recovery (%)
PE-1	0.83 ± 0.05 μg g^−1^	2.0 μg g^−1^	2.92 ± 0.16 μg g^−1^	104.5
PE-2	2.70 ± 0.14 μg g^−1^	2.0 μg g^−1^	4.65 ± 0.21 μg g^−1^	97.5
Drinking water	3.60 ± 1.06 ng mL^−1^	50.0 ng mL^−1^	54.4 ± 2.3 ng mL^−1^	101.6
Treated municipal Wastewater	4.60 ± 1.17 ng mL^−1^	50.0 ng mL^−1^	58.5 ± 0.10 ng mL^−1^	107.8

a Standard deviation (n = 6).

### 3.5 Comparison of the presented SA-LPME with other techniques in the literature

Numerous techniques have been documented for the extraction of phenolic compounds from various samples. The presented technique in this research was compared with other methods found in the literature, and the corresponding results are summarized in Table 6. The data in the table demonstrate that the proposed method in this study yields a favorable enrichment factor. The limit of detection obtained for the presented technique is comparable to some results reported in other literature. Notably, in this method, the 2-phenoxyethanol sample serves as the solvent, eliminating the need for additional solvents in the extraction of phenolic compounds from 2-phenoxyethanol samples. This characteristic renders the method distinctive, less hazardous, and environmentally friendly for the determination of phenol impurity in 2-phenoxyethanol. However, it is important to acknowledge that one limitation of this method, when compared to other techniques for phenol index determination, is the presence of phenol impurity in PE. This limitation can be addressed through purification using an alkaline solution.

**Table 6:** Comparison of the presented technique in this study with other methods for the detection of phenol.

Extraction method	LOD (ng mL^−1^)	RSD (%)	EF	Ref.
Salting out and vortex-assisted dispersive liquid-liquid microextraction based on solidification of floating organic drop microextraction	0.06	3.7	165	[23]
Salting out liquid-liquid extraction (SALLE) combined with dispersive liquid-liquid microextraction (DLLME)	0.15	4.8 to 7.2	78.12 to 82.53	[38]
Dispersive liquid-liquid microextraction (DLLME)	0.18	6 (n = 7)	920	[17]
Dispersive liquid-liquid microextraction combined with microvolume spectrophotometry	0.8	5.2 (n = 6)	700	[39]
Dispersive liquid-liquid microextraction coupled with the pressure-assisted electrokinetic injection	0.13	5.12 (n = 5)	61	[40]
Combination of dispersive liquid-liquid microextraction and smartphone-based colorimetric system	1.7	1.2 (n = 8)	33.3	[5]
Dispersive liquid-liquid microextraction based on terpineol-based hydrophobic deep eutectic solvent	0.38	5.4 (n = 6)	27	[19]
Molecular complex-based dispersive liquid-liquid microextraction	3.71	<9.1	–	[41]
Liquid-phase microextraction	0.4	9.0 (n = 3)	15	[42]
Salting-out assisted liquid phase microextraction	0.7	2.4	65	This research

^a^ Limit of detection.

^b^ Relative standard deviation.

## 4 Conclusions

Some results In conclusion, the performance of the SA-LPME technique coupled with spectrophotometric detection was assessed for the quantification of phenol impurity in 2-phenoxyethanol as well as total phenols in drinking water and treated municipal wastewater. This technique utilizes 2-phenoxyethanol as the extractant solvent, making it a novel, eco-friendly, low-toxicity, and cost-effective approach for phenol determination in 2-phenoxyethanol. The method involves the formation of a two-phase system, where PE droplets serve as the extractant phase, achieved by introducing NaCl to a homogeneous solution containing PE. Various parameters, including 4-AAP, buffer (pH = 10), hexacyanoferrate, and NaCl, were optimized to enhance the extraction performance. The reliability of the method was evaluated through the analysis of 2-phenoxyethanol, drinking water, and treated municipal wastewater samples. Notably, this technique offers a high enrichment factor, a low limit of detection, and satisfactory recovery. Considering the aforementioned advantages, this study presents a promising technique for the determination of phenolic compounds in diverse matrices.

Acknowledgments.

## Data statement

Authors agree to make data and materials supporting the results or analyses presented in their paper available upon reasonable request.

## CRediT authorship contribution statement

**Farideh Roustaei:** Formal analysis, Data curation, Conceptualization. **Majid Baghdadi:** Writing – review & editing, Supervision, Data curation, Conceptualization. **Azam Marjani:** Supervision, Methodology, Formal analysis. **Mohammad Alimoradi:** Methodology, Conceptualization.

## Declaration of competing interest

The authors declare that they have no known competing financial interests or personal relationships that could have appeared to influence the work reported in this paper.
